# Opportunistic Infections among People Living with HIV (PLHIV) with Diabetes Mellitus (DM) Attending a Tertiary Care Hospital in Coastal City of South India

**DOI:** 10.1371/journal.pone.0136280

**Published:** 2015-08-19

**Authors:** Poojary Indira, Papanna Mohan Kumar, Shenoy Shalini, Kulkarni Vaman

**Affiliations:** 1 Department of Microbiology, Apollo Hospitals, Chennai, Tamil Nadu, India; 2 Department of Community Medicine, Kasturba Medical College (Manipal University), Mangalore, Karnataka, India; 3 Department of Microbiology, Kasturba Medical College (Manipal University), Mangalore, Karnataka, India; Public Health Agency of Barcelona, SPAIN

## Abstract

**Background:**

HIV/AIDS and Diabetes Mellitus are the diseases’ known to supress cell mediated immunity and predispose patients for opportunistic infections. Hence, we conducted a study to compare the common opportunistic infections (OIs) between People Living with HIV with DM (PLHIV-DM) and PLHIV without DM (PLHIV).

**Methodology:**

PLHIV with DM and without DM (1:1) were prospectively included in the study from January 2011 to January 2012 at a tertiary care hospital in Mangalore city. Patients were classified as Diabetic if their fasting plasma glucose was ≥ 7.0mmol/l (126mg/dl) or 2–h plasma glucose was ≥11.1mmol/l (200mg/dl). Standard procedures and techniques were followed for diagnosis of OIs as per WHO guidelines. The data was entered and analyzed using Statistical Package for Social Sciences (SPSS) version 11.5.

**Findings:**

The study included 37 PLHIV with DM and 37 PLHIV without DM and both groups were treated with Anti-Retroviral Therapy (ART). The median age was 47 years (IQR: 41-55years) for PLHIV-DM as compared to 40 years (IQR: 35–45.5 years) for PLHIV (p<0.0001). PLHIV-DM had median CD4 counts of 245 (IQR: 148–348) cells/μl compared to 150(IQR: 70–278) cells/μl for PLHIV (p = 0.02). Common OIs included oral candidiasis (49% of PLHIV-DM and 35% of PLHIV); Cryptococcal meningitis (19% of PLHIV-DM and 16% of PLHIV); *Pneumocystis jiroveci* pneumonia (5% of PLHIV-DM and 18% of PLHIV); extra pulmonary tuberculosis (22% of PLHIV-DM and 34.5% of PLHIV); and Cerebral toxoplasmosis (11% of PLHIV–DM and 13.5% of PLHIV). Microbiological testing of samples from PLHIV- DM, *C krusei* was the most common Candida species isolated from 9 out of 18 samples. Out of six pulmonary TB samples cultured, four grew Non-tuberculosis mycobacteria (NTM) and two Mycobacterium tuberculosis complexes.

**Conclusions:**

Study did not identify any significant difference in profile of opportunistic infections (OIs) between PLHIV with and without Diabetes.

## Introduction

India is facing the double burden of communicable and non-communicable diseases, with the third largest number of People Living with HIV in the world. According to the “Joint United Nations programme on AIDS” report 2013, an estimated 210,000 people are infected by HIV in India [1]. Out of 1.21 billion population in the country, an estimated 40.9 million people are affected with Diabetes Mellitus. [2] [3]. There is adequate evidence from cohort studies regarding a higher incidence of glucose intolerance and DM among HIV infected individuals on combined Anti-Retroviral Therapy (ART) when compared to those not on ART [4].

Impaired glucose metabolism is implicated to be multifactorial, the role of protease inhibitors (PIs) and nucleoside reverse transcriptase inhibitors (NRTIs) leading to DM has been demonstrated by few prospective studies [5, 6]. Additionally, DM is known to supress the cell mediated immunity and increases the frequency of infections [7, 8].

According to a study conducted by Brown et al in America, diabetes mellitus was 4 times more prevalent in HIV positive individual on ART [9]. Though we have numerous studies addressing common opportunistic infection among PLHIV/AIDS, there is limited data available about opportunistic infections affecting PLHIV/AIDS with diabetes. Early recognition and treatment of OI in these individuals will improve their quality of life. Hence, this study was conducted with the objectives to study the pattern of opportunistic infections in those PLHIV with DM and differences in OI’s between those PLHIV, with and without DM.

## Materials and Methods

A prospective study was conducted at the Kasturba Medical College Hospital, Mangalore. This hospital has registered about 1500 AIDS patients and has a fully functioning ART centre supported by National AIDS control organization, Government of India. The study was conducted from January 2011-January 2012. PLHIVs’ with and without DM (1:1) were included in the study. PLHIV without DM where those individuals who had tested HIV positive without documented DM. Patients were classified as Diabetic if their fasting plasma glucose was ≥ 7.0mmol/l (126mg/dl) or 2–h plasma glucose was ≥11.1mmol/l (200mg/dl) [10]. Further, clinical staging of PLHIV with and without DM was done based on World Health Organization guidelines (WHO) [11]. PLHIV–DM admitted in the hospital were approached and a written informed consent was obtained. A list of PLHIV without DM admitted in the hospital during study period was obtained and every 5th patient was selected for the comparison group. Their laboratory reports were reviewed for data related to opportunistic infection. A semi structured pro-forma was used to collect socio demographic data, clinical features, CD4 counts and treatment. All opportunistic infections were diagnosed based on WHO guidelines [12]. Though most of the opportunistic infections diagnosed for PLHIV-DM had microbiological or histopathological evidence, there were cases of extra Pulmonary Tuberculosis and Cytomegalovirus (CMV) retinitis which were diagnosed clinically based on WHO criteria.

### Laboratory diagnosis

Due to resource constraints, laboratory testing was done on samples collected from PLHIV-DM based on the presenting symptoms and clinical features. Samples collected included sputum, oral swab, stool, urine, cerebrospinal fluid (CSF), lymph node aspirate& blood. All samples were collected following standard precautions in suitable sterile universal container. Blood samples were collected aseptically and inoculated into aerobic culture bottles only. The samples were transported to the laboratory immediately and processed without delay in a class II biological safety cabinet. Appropriate methods and procedures for diagnosing opportunistic infections were carried out at Kasturba Medical College, Microbiology laboratory which is a NABL (National Accreditation Board for testing and calibration of Laboratories) accredited laboratory. CSF samples were screened for pathogens using gram stain, ZN stain and India ink staining and cultured for pathogens. Sputum culture was done for patients suspected to have pulmonary tuberculosis on Lowenstein Jenson media. Mycobacterial speciation was done for this cultures using Immuno-chromatographic test called SD TB Ag MPT 64 (S.D. BIO STANDARD DIAGNOSTICS, South Korea). Speciation of candida was done using Chrome agar plates (HiMedia Laboratories).

Serological tests were performed for Cytomegalovirus (CMV) (IgM and IgG), Hepatitis B surface antigen (HBsAg), Herpes Simplex Virus (HSV1 & 2-IgM and IgG). Those patients with clinical suspicion of toxoplasmosis underwent radiological diagnosis followed by serological tests on serum samples (IgM and IgG). CD4 counts were collected from medical records as the test was performed at the ART centre as part of routine care.

### Data Analysis

The data was entered and analysed using Statistical Package for Social Sciences (SPSS) version 11.5 (SPSS Inc., 233 South Wacker Drive, 11th floor, Chicago, IL 60606–6412). The results were expressed as proportions and presented in the tables. Chi-square or Fisher exact test were used to examine the relation between categorical variable and Mann-Whitney U tests for relation between Medians.

### Ethical issues

Ethical clearance was obtained from Institutional ethics committee at Kasturba Medical College, Mangalore. Permission to conduct the study was obtained from the medical superintendent of the hospital.

## Results

The study included 37 PLHIV-DM and 37 PLHIV and all the cases and controls were on ART admitted in the hospital. Median age for PLHIV-DM group was 47 years (IQR: 41-55years) as compared to 40 years (35–45.5 years) for PLHIV group (p = <0.0001). Eighty one percent of PLHIV-DM and 76% PLHIVs’ were males (p = 0.57). Median duration following HIV diagnosis for PLHIV-DM was 3(1.5–6.5) years and 4 (2–7) years for PLHIV (p-0.32). The duration of Anti retro viral therapy was 3(1–5) years for both the groups (p-0.10). PLHIV-DM had median CD4 counts of 245 (148–348) cells/μl compared to 150(70–278) cells/μl for PLHIV (p = 0.02). Twenty seven percent of PLHIV-DM and 57% of PLHIVs were of WHO clinical stage IV (Table 1). Patients in both the groups were on combined ART drugs: Zudovudine/Stavidune with Lamivudine + Nevirapine/efavirenz [PLHIV-DM (34/37) and PLHIV (33/37)] or alternative first line drug Tenofovir with Lamivudine + Nevirapine/efavirenz [PLHIV-DM (3/37) and PLHIV (4/37)].

**Table 1:** Demographic and Clinical Profile of PLHIV-DM and PLHIV.

Variables		PLHIV with DM n (%)	PLHIV without n (%)	P value
**Age group in years**	<40	9(24)	22(59.5)	0.002
	≥40	28(75)	15(40.5)	
**Median age in years(IQR)**		47(41–55) years	40(35–45.5) years	<0.0001
**Gender**	Male	30(81)	28(76)	0.57
	Female	7 (19)	9(24)	
**Duration of HIV positivity**	<5 years	22(59.5)	21(57.0)	0.81
	≥5 years	15(40.5)	16(43.0)	
**Median duration of HIV Positivity in years (IQR)**		3 (1.5–6.5) years	4 (2–7) years	0.32
**Duration of ART**	<5 years	23(65.7)	26(70.0)	0.46
	≥5years	12(34.3)	11(30.0)	
**Median duration of ART(IQR) years**		3(1–5) years	3(1.5)years	1.00
**Duration of DM**	<5 years	24(65)	-	
	≥5years	14(35)	-	
**CD4 counts (cells/μl)**	<200	15(40.5)	22(59.5)	0.10
	≥200	22(59.5)	15(40.5)	
**Median CD4(IQR) cells/μl**		245 (148–348)	150(70–278)	0.02
**WHO Clinical Staging**	Stage I &II	13(35)	14(38.0)	0.001
	Stage III	14(38)	2(5.0)	
	Stage IV	10(27)	21(57.0)	

All the PLHIV with DM had one or the other form of opportunistic infections of which 12 patients had more than one type of opportunistic infections as compared to 34 patients in PLHIV group with at least one opportunistic infections. Fungal infections were identified among 29(78.4%) PLHIV-DM patients compared to 19(51%) of PLHIV patients (p = 0.03), Bacterial infections was seen among 19(51.4%) compared to 21(57%) of PLHIV patients (p = 0.81), protozoal infections was seen among 10(27%) when compared to 11(30%) of PLHIV patients (p = 1.0) and viral infections in 5(13.5%) PLHIV-DM patients compared to 5(13.5%) of PLHIV patients (p = 1.0).

The most common clinically diagnosed opportunistic infections was oral candidiasis among 49% of PLHIV-DM and 35% of PLHIV. Cryptococcal meningitis was diagnosed among 19% of PLHIV-DM and 16% of PLHIV and *Pneumocystis jiroveci* pneumonia was diagnosed among 5% of PLHIV-DM compared to 18% of PLHIV. Among bacterial infections extra pulmonary tuberculosis was diagnosed among 22% of PLHIV-DM and 34.5% of PLHIV. Cerebral toxoplasmosis was diagnosed among 11% of PLHIV–DM compared to 13.5% of PLHIV (Table 2).

**Table 2:** Common Opportunistic infections confirmed clinically or by culture among patients with PLHIV-Diabetic and PLHIV.

Opportunistic infections	PLHIV with DM n(%)	PLHIV without DM n(%)	p value*
**Bacterial infections** ^#^			
Extra pulmonary Tuberculosis	8(22)*	12(34.5)	0.43
Pulmonary tuberculosis	6(16)	6(16)	1.00
*E*.*coli* infection	4(11)	3(9)	1.00
*Pseudomonas aeruginosa* infection	2(5)	3(9)	1.00
*S aureus* infection	1(3)	2(5)	1.00
**Fungal infections** ^#^			
Oral Candidiasis	18(49)	13(35)	0.24
Candidiasis (other sites)	7(19)	3(8)	0.30
Cryptococcal meningitis	7(19)	6(16)	1.00
*Pneumocystis jiroveci* pneumonia	2(5)	7(18)	0.15
*Aspergillus flavus* infection	1(3)	-	-
**Protozoal Infections** ^#^			
Cerebral Toxoplasmosis	4(11)	5(13.5)	1.00
Cryptosporidiosis	3(8)	-	-
Giardia infection	-	1(3)	-
**Viral infections** ^#^			
CMV retinitis^**^	2(5)	3(8)	1.00
*Herpes simplex* infection	1(3)	1(3)	1.00
Hepatitis B infection	2(5)	1(2.7)	1.00
Herpes zoster infection	-	2(5)	-
Molluscum contagiosum	-	1(3)	-

^#^Pearsons Chi square and Fisher exact tests

* 3 out of 8 cases diagnosed histo-pathologically

**diagnosed clinically.

Of 18 samples collected from patients with candida infections, *Candida krusei* species was isolated in 9/37, *C albicans* in 5/37, *C tropicalis* in 3/37 and *C glabrata* in 1/37. Out of the 6 sputum samples cultured for pulmonary TB, four samples were identified as Non-tuberculosis mycobacteria (NTM) and two Mycobacterium tuberculosis complexes (Table 2).

All OI s in PLHIV-DM and PLHIV had Microbiological or Histopathological confirmation except out of the 8 samples from suspected cases of extra pulmonary tuberculosis among PLHIV-DM, 3 samples did not show any growth even after prolonged incubation hence speciation was not done for extra pulmonary samples. All cases of CMV retinitis i.e 2 cases among PLHIV and 3 cases among PLHIV-DM were diagnosed clinically.

## Discussion

This is one of the first studies to the best of our knowledge reporting common opportunistic infections among the subgroup of patients with PLHIV with DM compared with PLHIV without DM from South India. The study detected fungal infections among majority of our patients followed by protozoal and bacterial infections in both groups. Majority of patients in both groups were males, with a significant difference in age between the groups; PLHIV-DM being around 47 years, comparable with the age group affected by type II DM [13]. Despite both groups having been HIV positive and received ART for the same duration of time, those PLHIV diagnosed to have DM had better CD4 count. Among PLHIV with DM being a diabetic could have led to more frequent follow up visits, which could have indirectly improved adherence to ART leading to higher CD4 counts or PLHIV with DM may have complications of OIs which are less likely to be successfully treated on outpatient hence, these patients may have high CD4 counts on admission. Influence of the above factors on CD4 levels need to be further studied.

Oral candidiasis was the most common opportunistic infection in both the groups, being higher in those with DM. This difference could be because candida is the most common opportunistic infection among WHO clinical Stage I, II and III patients and most of PLHIV with DM were in these stages or presence of two immunosuppressive diseases could have led to high proportion of candida infection among the diabetic group. We isolated *Candida krusei* from samples collected from PLHIV-DM, a finding that is not uncommon. However, some studies have also shown non-albicans species as being frequently isolated from both HIV/AIDS and patients with diabetes [14–17]. Cryptococcal meningitis was the next common fungal infection identified among PLHIV-DM and PLHIVs in our study. Studies on Cryptococcal infection in AIDS patients from different parts of India have revealed varying results. The study conducted among AIDS patients by Manoharan G et al in Perundurai [18] reported this infection in 34.8% patients, while Saldana D et al at Mangalore reported 8.2%patients [19] and Ayyagari in Northern India reported 5.6%cases [20]. In the current study, it was identified in about 19% of PLHIV–DM cases, falling within this wide range of findings, but almost twice as much, when compared to the findings of the study conducted by Saldana et al [19] in the same setup.

Tuberculosis (TB) is the most common opportunistic infection leading to 25% mortality among AIDS patients [21] and Diabetes is a risk factor for tuberculosis [22]. Pulmonary tuberculosis was seen equally among both groups, while that extra-pulmonary tuberculosis was diagnosed in a slightly higher number among PLHIV patients when compared to PLHIV-DM patients. The difference could be due to significant difference in CD4 counts between the groups, with the latter having a higher CD4 count. Among pulmonary TB isolates, NTMs’ were identified in four out of six patients when compared to Mycobacterium tuberculosis complex (two out of six patients). These findings are similar to findings of a study done by Horsburgh C in Atlanta, in PLHIV (AIDS) [23]. A study among HIV positives (non AIDS) individuals has shown that NTMs are associated with fewer cases of pulmonary infections when compared to M tuberculosis complex [24].

A slightly higher number of PLHIVs were affected by cerebral toxoplasmosis when compared to PLHIV-DM (13.5% Vs 11%). The findings in this study are higher than some Indian studies (Sharma et al in north India among PLHIV- 3.7%) [25]. *Pneumocystis jerovecii* pneumonia (PCP pneumonia) is the most common OI in western world [26]. Studies related to PCP pneumonia from the Indian subcontinent are very limited. One such study done by Udwadia et al in Mumbai showed 13% of HIV admissions were due to PCP pneumonia [27]. Eighteen percent of PLHIVs in our study had PCP as compared to 5% in PLHIV-DM. The difference in PCP and cerebral toxoplasmosis rates between PLHIVs with and without DM could be attributed to significant difference in CD4 counts in our study.

The study was limited to a very small group of patients and was from a single tertiary care hospital. Factors such as adherence to ART, type of diabetic medication received by those with DM, and Glycaemic control were not studied. Inclusion of the above factors would make interpretation of findings easier, and these factors need to be addressed in subsequent studies.

## Conclusion

The group of patients with DM were older. All the PLHIV with DM had at least one OI with one third of them having more than one OIs. Although PLHIV-DM had higher proportion of oral candidiasis and Cryptococcal meningitis than their counterparts, this study did not identify any significant difference in profile of opportunistic infections (OIs) between PLHIV with and without Diabetes.
